# Assessing the Nomological Network of the South African Personality Inventory With Psychological Traits

**DOI:** 10.3389/fpsyg.2021.727848

**Published:** 2021-10-11

**Authors:** Carin Hill, Jan Alewyn Nel, Leon T. de Beer, Velichko H. Fetvadjiev, Lyle I Stevens, Monique Bruwer

**Affiliations:** ^1^Department of Industrial Psychology and People Management, College of Business and Economics, University of Johannesburg, Johannesburg, South Africa; ^2^Department of Human Resource Management, University of Pretoria, Pretoria, South Africa; ^3^WorkWell Research Unit, North-West University, Potchefstroom, South Africa; ^4^Department of Social Psychology, University of Amsterdam, Amsterdam, Netherlands

**Keywords:** South African Personality Inventory, nomological network, psychological traits, general anxiety, work locus of control, psychological well-being, cultural intelligence

## Abstract

The purpose of this study was to expand internal construct validity and equivalence research of the South African Personality Inventory (SAPI), as well as to investigate the nomological validity of the SAPI by examining its relationship with specific and relevant psychological outcomes. The internal and external validity of the SAPI was assessed within three separate samples (*N* = 936). Using the combined data from all three samples, Exploratory Structural Equation Modelling (ESEM) indicated that the six-factor SAPI model fit proved to be excellent. Measurement invariance analyses showed that the SAPI dimensions in the ESEM model were invariant across gender and race groups. Next, two separate studies explored the associations of the SAPI factors with relevant psychological outcomes. An ESEM-within-CFA (set ESEM) method was used to add the factors into a new input file to correlate them with variables that were not part of the initial ESEM model. Both models generated excellent fit. In Study 1, psychological well-being and cultural intelligence were correlated with the SAPI factors within a sample of students and working adults. All of the psychological well-being dimensions significantly correlated with the SAPI factors, while for cultural intelligence, the highest correlations were between Meta-cognition and Openness and Meta-cognition and Positive Social-Relational Disposition. In Study 2, work locus of control and trait anxiety was correlated with the SAPI factors within a sample of adults from the general South African workforce. Work Locus of Control correlated with most factors of the SAPI, but more prominently with Positive Social-Relational Disposition, while Neuroticism correlated strongly with trait anxiety. Finding an appropriate internal structure that measures personality without bias in a culturally diverse context is difficult. This study provided strong evidence that the SAPI meets the demanding requirements of personality measurement in this context and generated promising results to support the relevance of the SAPI factors.

## Introduction

Indigenous measures of personality have been developed over recent decades to meet local needs in various non-Western cultures and to reduce the prevailing reliance on imported instruments (Fetvadjiev and Van de Vijver, 2015; Cheung and Fetvadjiev, 2016; Church, 2017). Early indigenous research has devoted extensive attention to the specific cultural (emic) interpretation of local personality constructs. More recent lines of research have sought to expand the culturally specific focus of early studies by applying a broader comparative approach that includes both local and presumed universal (etic) elements in a combined emic–etic approach (Cheung et al., 2011). This approach is characterised by direct comparisons of indigenous instruments to universal concepts, an assessment of indigenous measures’ cross-cultural replicability, and examining the predictive value of indigenous instruments for locally relevant outcomes. Notably the second and third aspect have received relatively less attention in the literature (Church, 2017). The present study aims to examine the predictive value of an indigenously developed instrument, the South African Personality Inventory (SAPI; Fetvadjiev et al., 2015), for consequential outcome variables in the domains of cultural intelligence, well-being, and personal growth across three multi-ethnic samples in South Africa.

Personality is known to be related to a range of consequential life outcomes (Ozer and Benet-Martínez, 2006). Evidence has also started accumulating that indigenous or emic–etic measures have a role to play in predicting relevant outcomes. For example, Katigbak et al. (2002) found that Philippine personality scales were associated with various self-reported behaviours and attitudes; the indigenous scales offered improved prediction over and above a Big Five instrument notably for praying. Based on the extensive research programme on the Chinese Personality Assessment Inventory (CPAI), Cheung et al. (2013) reported that the CPAI was related to behaviours indicating variety of interests (e.g., learning languages), variety in social networks (e.g., seeking and giving advice), and interpersonal behaviours with family and friends (e.g., quarrelling and gift-giving). It is worth noting that these associations were observed both in Asian countries (China, South Korea, and Japan) and in the United States, highlighting the emic–etic aspects of the CPAI. In one of the few indigenous studies outside Asia so far, Burtăverde et al. (2018) found that a Romanian indigenous personality instrument explained variance in social adaptation (e.g., career satisfaction), risky social behaviours (e.g., driving fines), and status-striving (e.g., materialism). These research programmes illustrate the value of examining the nomological networks of indigenously derived measures by assessing their associations with relevant criterion variables. Still, this field of research has remained limited and has mostly been confined to Asian samples. The present study aims to advance the field by analysing important criterion variables in the nomological network of an African-derived instrument, the SAPI.

## The South African Context and the SAPI

In South Africa, a markedly multicultural society, the government requires that psychological assessments comply with specific legislation, including the Employment Equity Act (EEA) Section 8 (Act 55 of 1998). The EEA states that psychological assessments need to be scientifically shown to be valid and reliable, fair to all employees, and should not discriminate based on language, race, gender, or culture in any way (see The Republic of South Africa, 1998).

The SAPI project’s goal has been to provide South Africa with a personality model that takes into account the implicit concepts of personality found across the 11 official spoken languages (Afrikaans, English, isiNdebele, isiXhosa, isiZulu, Northern Sotho, Setswana, Siswati, Southern Sotho, Tshivenda, and Xitsonga) and that substantiates a psychometrically sound inventory in terms of reliability and validity (Nel et al., 2012; Fetvadjiev et al., 2015). The SAPI was initially conceptualised as a nine-factor model that included Conscientiousness, Emotional Stability, Extraversion, Facilitating, Integrity, Intellect, Openness, Relationship Harmony, and Soft-Heartedness (Nel et al., 2012). Building on this conceptual model, Fetvadjiev et al. (2015) found a factor structure that contains 18 facet scales representing six factors labelled Conscientiousness, Extraversion, Neuroticism, Openness, Negative Social-Relational Disposition, and Positive Social-Relational Disposition. *Conscientiousness* is defined as an individual’s orientation toward success, precision, and conventionalism, while *Extraversion* is an individual’s tendency toward spontaneous interactions while entertaining others through jokes and stories. The *Neuroticism* factor represents the tendency to be impulsive and have fluctuating emotions, whereas the *Openness* factor describes the quality of being well-informed, rational, and a progressive thinker. The two social-relational factors address how a person typically approaches their relationships with others: *Negative Social-Relational Disposition* describes the extent to which a person typically approaches relations with others in a contentious manner, whereas *Positive Social-Relational Disposition* illustrates a person’s inclination toward a positive approach in managing relations with others. Fetvadjiev et al. (2015) established that the SAPI factors were equivalent across various ethnic groups and correlated with impression-management qualities of social desirability while producing weak correlations with deceitful qualities of social desirability. The SAPI’s social-relational factors remained relatively distinct when compared to measures of the Big Five (see Valchev et al., 2014; Fetvadjiev et al., 2015). Morton et al. (2019) used a 20-facet version of the SAPI and confirmed the same six-factor structure. Finally, the SAPI model has been replicated in two cultural groups in New Zealand, where the SAPI was found to add incremental value above a Big Five instrument in the prediction of family orientation and well-being (Fetvadjiev et al., 2021).

### External Construct Validity

While it is important for newly developed measuring instruments to produce valid and reliable factors, construct validity-related evidence is essential to ensure the actual use of such an instrument in the relevant field (Ziegler et al., 2013). Cronbach and Meehl (1955) introduced the “nomological network” in 1955, stating that such an interlocking system provides researchers with the opportunity to learn more about and enrich a theory-based construct through certain methodological principles which allow for the scientific confirmation of the construct validity of psychological tests. These principles include amongst others that constructs should exhibit frequent lawful relationships with other constructs, and lawful relationships include establishing connections between observable manifestations, between theoretical and observable constructs, or between various theoretical constructs – either statistically or deterministically (Cronbach and Meehl, 1955; Belkhamza and Hubona, 2018). Furthermore, a nomological network provides evidence on how a construct predicts outcome criteria and increases the definiteness of the factors of the theoretical construct (Cronbach and Meehl, 1955; Zettler et al., 2020).

Forming a nomological network is, therefore, a significant way to assess construct validity, and it involves both the internal and external examination of a particular construct (Cronbach and Meehl, 1955; Byrne, 1984). Internal examination studies the relationships among the various construct facets and indicates the legitimacy and replicability of the results, while external examination studies the relationships between the construct and other presumably mutually exclusive constructs (Byrne, 1984; Ziegler et al., 2013). The current study aimed to expand the investigation into the psychometric properties of the SAPI through (1) examining the internal construct validity and equivalence of the SAPI, and (2) examining the external construct validity of the SAPI by way of establishing a nomological network between the SAPI factors and relevant psychological outcomes.

External construct validity within the current study was assessed by examining to what extent the SAPI factors are related to other psychological traits that should be theoretically related (concurrent validity), as well as to what extent the SAPI factors are different from other psychological traits that should be theoretically unrelated (discriminant validity) (Cronbach and Meehl, 1955). Psychological traits can be defined as the “…relatively stable or enduring individual differences in thoughts, feelings and behaviour…” (Church, 2000; p. 651) and a literature search on PsycINFO regarding meta-analytical studies of the relationship between personality and various psychological traits produces a vast amount of research papers. For example, the major factors of personality have been linked with psychological traits such as anxiety (Kotov et al., 2010; McKinney et al., 2021), humour (Mendiburo-Seguel et al., 2015), mindfulness (Giluk, 2009; Ortet et al., 2020), narcissism (Grijalva and Newman, 2015), subjective well-being (Anglim et al., 2020; Ortet et al., 2020), and values (Fischer and Boer, 2015; Nei et al., 2018), to name but a few (see also Ozer and Benet-Martínez, 2006). Within the South African context, various personality traits have been linked with psychological traits such as anxiety (Van Jaarsveld and Schepers, 2007), social adjustment (Papageorgiou and Callaghan, 2018), emotional competence (Coetzee et al., 2006), cultural intelligence (Nel et al., 2015), locus of control (Schepers and Hassett, 2006; Van Wyk et al., 2009), psychological well-being (Jones et al., 2015), and self-esteem (Coetzee et al., 2006). The majority of previous research has used the established Big Five model. The present study broadens this scope by examining several important correlates of an indigenously derived, emic–etic instrument in South Africa. Four external criterion variables were included in the current study to assess the external construct validity of the SAPI: cultural intelligence, general anxiety, psychological well-being, and work locus of control.

## Overview

In this study, we used three separate samples to examine the SAPI’s internal and external validity. Sample A was derived using purposive non-probability sampling and included industrial psychologists, intern industrial psychologists, psychometrists, and students in industrial psychology. Sample B was based on non-probability convenience sampling design to collect data from South Africa’s general workforce. The participants’ work contexts were varied and included financial, accounting and banking industries, sports and medicine, fast-moving consumer goods, law, events, education, engineering, marketing, IT, and non-profit organisations. Sample C was also obtained using a non-probability convenience sampling strategy and included students in a higher education institution in South Africa. The demographical details for each sample can be found in Table 1.

**TABLE 1:** Sample characteristics.

Sample	Total *n*	Gender			Race				
		Men	Women	Missing	Black	Coloured	Indian	White	Missing
A	400	152	248	0	62	45	8	284	1
B	422	146	269	7	80	34	43	244	21
C	114	43	71	0	15	17	3	79	0
Total	936	341	588	7	157	96	54	607	22

A combined investigation including all three samples was done to examine the internal validity and measurement invariance (based on gender and ethnicity). The external validity was examined in two separate studies by determining the relations of the SAPI factors with various psychological traits. Study 1 (using Sample A) focused on cultural intelligence (CQ) and psychological well-being (PWB) as criterion variables. In Study 2 (using Sample B), we directed our focus to trait anxiety and to work locus of control (WLC) as an important aspect of functioning in the work environment.

### Preliminary Study: Psychometric Properties of the SAPI

Since the purpose of the SAPI project included developing an assessment measure that could be used across ethnic groups within South Africa, it is important to investigate the construct equivalence of the inventory across groups and samples. Fetvadjiev et al. (2015) determined that the equivalence of the six-factor structure of the SAPI was at least fair, and even very good in most comparisons across the four official ethnic groups within South Africa (Black^1^, Coloured^2^, Indian, and White), as well as within a replication study amongst a sample containing only Black and White participants. Fetvadjiev et al. (2015) study was done only amongst university students and adults from security or insurance companies and used the relatively lenient framework of exploratory factor analysis. Morton et al. (2018) investigated the factor structure among various industries and managerial positions using a more stringent structural-equation-modelling approach and found the equivalence of the factor structure of the SAPI to be very good. However, Morton et al. (2018) sample size was relatively small (*n* = 313), and therefore the further investigation of the psychometric properties of the SAPI in a larger and more varied sample is warranted. Building on this previous work, the current study examines the SAPI’s properties in a large, multiethnic sample employing a structural-equation-modelling approach. We examine the SAPI’s measurement equivalence across three of the country’s four ethnic groups (Blacks, Coloureds, and Whites) as well as both genders.

Investigations of construct equivalence given a previously established factor structure are typically done using confirmatory factor analysis (CFA; Van de Vijver and Leung, 2021). However, CFA presents certain limitations regarding the latent variable measurement specification that warrant using a different approach, namely exploratory structural equation modelling (ESEM; Asparouhov and Muthén, 2009; Van de Vijver and Leung, 2021). There are two main constraints of CFA: (1) it has a stringent requirement of zero cross-loadings that causes the data to produce misfitting models, and subsequent researchers tend to modify their models extensively in search of model fit; and (2) the consequence of misspecified zero loadings in CFA is the distortion of factors, over-estimated factor correlations, and distorted structural relationships (Asparouhov and Muthén, 2009). ESEM, on the other hand, incorporates some of “the best features of exploratory factor analysis (EFA), confirmatory factor analysis (CFA), and structural equation modeling (SEM)” (Marsh et al., 2013, p. 2). While CFA requires the rigorous adherence to item cross-loadings fixed to zero, ESEM allows the estimation of item cross-loadings. As such, theoretically, the latent factor inter-correlations of independent personality factors will be considerably smaller compared to CFA-estimated correlations (Ginns et al., 2014). Therefore, in the current study, the model fit of the six-factor SAPI model was tested using ESEM.

#### Method

The SAPI was administered to 936 students and working adults in Samples A, B, and C. Apart from meeting the general target sample descriptions of the respective sample (working adults and students in the fields of industrial psychology and psychometrics in Sample A; working adults from the general work force in Sample B; students in Sample C), participants had to be 18 years or older to complete the questionnaires. Research proposals concerning the studies were presented to research committees at the various supervising universities, and ethical clearance was granted for each study. Participation was voluntary, and the purpose of the research was clearly explained. Each participant was provided with a letter of consent, and ethical aspects such as confidentiality were explained and assured, as well as the option to withdraw at any given moment. Due to its small sample size, the Indian race group was excluded from the current analyses.

The SAPI version used in this study consisted of 146 items grouped into 19 facet scales, representing the six SAPI factors. The version used in this study was a preliminary version of the SAPI that was adapted in the articles by Fetvadjiev et al. (2015) and Morton et al. (2019). The responses are provided on a Likert scale that ranges from 1 (strongly disagree) to 5 (strongly agree). The facet scores were used as indicators of the factors in this study. The ESEM analyses were executed using robust maximum likelihood estimation (MLR) in Mplus 8.6 (Muthén and Muthén, 2021). To assess the measurement invariance of the model, we tested the configural, metric, and scalar invariance. Due to the sensitivity of chi-square to sample size, we used the rule of thumb for maximum change in CFI (0.01), SRMR (0.030), and RMSEA (0.015) (Chen, 2007).

#### Results

The six-factor model of the SAPI was fitted to the observed data using ESEM. The fit of the six-factor model proved to be excellent: χ*^2^* = 333.77 (*df* = 72, *p* < 0.001), RMSEA = 0.062 (90% CI: 0.056, 0.069), CFI = 0.975, TLI = 0.942, SRMR = 0.015. The Cronbach’s alpha reliabilities of the facets ranged between 0.71 and 0.89, with the exception of Straightforwardness (0.58) and Deceitfulness (0.60), both of which have only three items. At the factor level, the following reliability coefficients were found: Conscientiousness (0.93), Extraversion (0.89), Neuroticism (0.84); Openness (0.88); Negative Social-Relational Disposition (0.90); Positive Social-Relational Disposition (0.96). Table 2 provides the correlations between the factors. The results of the measurement invariance testing showed that the SAPI structure was invariant at the scalar level across both gender and race groups according to the adopted cut-off criteria (see Table 3).

**TABLE 2:** Correlation matrix of the latent variables.

	*M*	SD	1	2	3	4	5
Conscientiousness	4.07	0.79	–				
Extraversion	3.78	0.90	0.30	–			
Neuroticism	2.57	0.98	−0.30	−0.01	–		
Openness	4.01	0.78	0.56	0.55	−0.24	–	
Negative Social-Relational Disposition	2.00	0.92	−0.30	−0.01	0.34	−0.09	–
Positive Social-Relational Disposition	3.98	0.72	0.65	0.58	−0.15	0.65	−0.28

*All displayed correlations above and below −0.01 are significant at *p* < 0.05 or lower.*

**TABLE 3:** Results of the measurement invariance testing.

Model group: gender	χ ^2^	*df*	CFI	Δ CFI	RMSEA	Δ RMSEA	SRMR	Δ SRMR
Configural invariance	402.39	144	0.976	–	0.062	–	0.017	–
Metric invariance	484.91	222	0.976	0.000	0.050	0.012	0.045	−0.028
Scalar invariance	496.74	235	0.976	0.000	0.049	0.001	0.044	0.001

**Model group: ethnicity**	**χ ^2^**	***df***	**CFI**	**Δ CFI**	**RMSEA**	**Δ RMSEA**	**SRMR**	**Δ SRMR**

Configural invariance	414.80	216	0.980	–	0.057	–	0.018	–
Metric invariance	631.20	372	0.974	0.006	0.049	0.008	0.044	−0.026
Scalar invariance	675.09	398	0.972	0.002	0.049	0.000	0.047	−0.003

*Gender, male and female; ethnicity, black, coloured, and white.*

### Study 1: The SAPI, Cultural Intelligence, and Psychological Well-Being

Construct validation research focuses on finding empirical confirmation that certain hypothesised relationships exist within a construct’s nomological network (Byrne, 1984). Both CQ and PWB are known to be related to personality (Ryff, 1989; Ang et al., 2006; Grant et al., 2009; Cheung et al., 2012; Nel et al., 2015) and were examined as relevant outcomes.

Cultural intelligence is defined as an individual’s ability to function in a multicultural setting or situation (Earley and Ang, 2003). According to Earley and Ang (2003) and Ang et al. (2006), CQ consists of four dimensions: Behavioural CQ (an individual’s ability to act appropriately following multicultural aspects, such as values and beliefs of different cultures); Cognitive CQ (an individual’s knowledge of multicultural aspects); Meta-cognitive CQ (an individual’s thought processes in order to understand cultural contexts); and Motivational CQ (the amount of energy an individual invests in understanding multicultural aspects). Research focusing on the relationship between personality and CQ has increased over the last decade (see Ott and Michailova, 2018). The four CQ dimensions tend to correlate with Agreeableness mostly, but also with Conscientiousness, Extraversion, and Openness (Huff et al., 2014; Li et al., 2016; Presbitero, 2016; Shu et al., 2017; Wang et al., 2019; Camargo et al., 2020). Li et al. (2016) found correlations between Emotional Stability and Behavioural CQ, Meta-cognitive CQ, and Motivational CQ. Huff et al. (2014) identified relationships between Intellect and Cognitive CQ, Meta-cognitive CQ, and Motivational CQ, whereas Shu et al. (2017) established that the HEXACO’s Honesty-Humility factor significantly correlated with Meta-cognitive CQ and Motivational CQ. To date, only Nel et al. (2015) provided evidence on the ability of SAPI factors and facets using the initially conceptualised nine-factor SAPI model to predict CQ. Their study concluded that Intellect and Facilitating predicted Meta-cognitive CQ; Soft-heartedness, Facilitating, and Extraversion predicted Motivational CQ; while Soft-heartedness and Conscientiousness predicted Behavioural CQ. CQ is of central importance for functioning in multicultural contexts such as South Africa. It is thus highly relevant to examine the role of local personality measures, particularly the SAPI with its emphasis on social-relational aspects, in accounting for individual differences in CQ.

Psychological well-being, in turn, refers to individuals’ need to function optimally, realise attributes and talents unique to themselves, and focus on identity, purpose and meaning, and relations to others (Ryff and Keyes, 1995; Ryff and Singer, 1996). Ryff (1989) model of PWB consists of six core factors: Autonomy (going about following one’s standards rather than the opinions of others); Environmental Mastery (participation in external activities); Personal Growth (to advance in knowledge, skills, and potential); Positive Relations with Others (the presence of close relationships with others in one’s life); Purpose in Life (having a sense of determination and significance in one’s life); and Self-acceptance (maintaining a positive attitude toward oneself). Correlational relationships have been established between the six PWB factors and personality traits. Neuroticism and negative affect tend to be negatively correlated with all of the PWB factors (Schmutte and Ryff, 1997; Burns and Machin, 2010; Jones et al., 2015; Anglim and Grant, 2016), while Schmutte and Ryff (1997) found no correlation between negative effect and Personal Growth. Extraversion and positive affect also tend to be positively correlated with all PWB factors (Schmutte and Ryff, 1997; Burns and Machin, 2010; Anglim and Grant, 2016), although Jones et al. (2015) found no correlation between extraversion and Autonomy. Openness has been found to be correlated with all PWB factors except Environmental Mastery, and Agreeableness, with all factors except Autonomy (Anglim and Grant, 2016). Lastly, Conscientiousness positively correlated with all of the PWB factors (Jones et al., 2015; Anglim and Grant, 2016).

It was speculated that the components of the SAPI would relate to certain CQ and PWB dimensions, contributing to convergent validity. Based on the previous research on CQ, it was expected that CQ as a whole would be most systematically related to the Positive Social-Relational domain, conceptually related to Agreeableness. Furthermore, associations between Openness and the cognitive and motivational aspects of CQ could be expected. For example, people who possess the Openness characteristics of being well-informed, a quick learner, adaptable, articulate, innovative, and perceptive would in all likelihood be more knowledgeable of customs in cultures different from their own and have a higher level of deliberate cultural consciousness when interacting with people from different cultures (i.e., high on Cognitive CQ and Meta-cognitive CQ). With respect to PWB, the most consistent relationships could be expected for Neuroticism and Conscientiousness, with more varied relationships for the other factors. It was also anticipated that a person who tends to be accommodating and loyal, compassionate and encouraging, as well as understanding and considerate (high on Positive Social-Relational Disposition), would probably also rank high on having close relationships with others in one’s life (positive relations with others). Finally, people who tend to be indiscreet and deceitful, who tend to exclusively focus on their own needs and see themselves as more important than others (Negative Social-Relational Disposition), will presumably have less satisfying interpersonal relationships and less engagement with other cultures (hence, negative relationships with CQ and PWB aspects).

#### Method

The SAPI, a cultural intelligence measure, and a psychological well-being measure were administered to 400 students and working adults in Sample A. The SAPI factor scales had the following values of Cronbach’s alpha: Conscientiousness (0.93), Extraversion (0.88), Neuroticism (0.81), Openness (0.86), Negative Social-Relational (0.89), and Positive Social-Relational (0.96).

A 20-item Cultural Intelligence Scale developed by Van Dyne et al. (2015) was used. The scale consists of four factors labelled Meta-cognitive CQ (4 items; e.g., “I am conscious of the cultural knowledge I apply to cross-cultural interactions”), Cognitive CQ (6 items; “I know the arts and crafts of other cultures”), Motivational CQ (5 items; “I enjoy interacting with people from different cultures”), and Behavioural CQ (5 items; “I vary the rate of my speaking when a cross-cultural situation requires it”). Each of these dimensions is measured on a five-point response scale ranging from 1 (strongly disagree) to 5 (strongly agree). The internal consistency of the CQ constructs within a South African sample (Nel et al., 2015) were more than adequate, with alpha coefficients ranging between 0.82 (Motivational CQ) and 0.91 (Behavioural CQ).

We used the Psychological Well-being Scale (PWBS) developed by Ryff (1989), which consists of 84 items representing the six dimensions. Each item within the dimensions is answered on a five-point response scale ranging from 1 (strongly disagree) to 5 (strongly agree). Studies using the 84-item version of the PWBS found acceptable Cronbach’s alpha coefficients ranging between 0.77 and 0.93 for the six dimensions (Ryff, 1989; Van Dierendonck, 2004; Davidson, 2006). The Cronbach’s alpha coefficients of the CQ and PWB measures in the current study are presented in Table 4.

**TABLE 4:** Correlations of the latent variables of Study 1.

	*M*	SD	α	C	E	N	O	NSR	PSR
Meta-cognition CQ	3.67	0.68	0.79	0.18	0.15	−0.24	0.38	−0.08	0.38
Cognitive CQ	3.04	0.75	0.84	0.10	0.17	−0.16	0.20	−0.05	0.14
Motivational CQ	3.59	0.71	0.81	0.07	0.20	−0.25	0.37	−0.10	0.26
Behavioural CQ	3.24	0.79	0.84	0.04	0.15	−0.12	0.28	−0.01	0.25
Autonomy	4.05	0.56	0.81	0.30	0.13	−0.60	0.34	−0.31	0.24
Environmental mastery	4.08	0.54	0.84	0.56	0.20	−0.66	0.29	−0.37	0.42
Purpose in life	4.37	0.55	0.84	0.61	0.20	−0.53	0.35	−0.38	0.46
Personal growth	4.45	0.51	0.83	0.36	0.31	−0.46	0.63	−0.27	0.50
Positive relations	4.28	0.55	0.82	0.31	0.51	−0.37	0.34	−0.33	0.47
Self-acceptance	4.22	0.60	0.87	0.42	0.19	−0.66	0.26	−0.32	0.32

*All displayed correlations below −0.10 and above 0.10 are significant at *p* < 0.05 or lower.*

*C, conscientiousness; E, extraversion; N, neuroticism; O, openness; NSR, Negative Social-Relational Disposition; PSR, Positive Social-Relational Disposition.*

#### Results and Discussion

The six-factor model of the SAPI was fitted to the observed data using ESEM. The fit of the six-factor model proved to be excellent: χ^2^ = 202.04 (*df* = 72; *p* < 0.001), RMSEA = 0.067 (90% CI: 0.056, 0.078), CFI = 0.969, TLI = 0.928, SRMR = 0.018. Then an ESEM-within-CFA (set ESEM) method was used to add the SAPI factors into a new input file in order to be able to correlate the SAPI factors with variables that were not part of the ESEM model. This specification allows for the retention of the ESEM parameters for the SAPI model whilst not allowing any cross-loadings from the other variables’ items in the model, which retain their traditional CFA structure. For the outcome variables, latent variables were specified by using the composite score as a single indicator for the respective latent variable, with the residual variance constrained to 1-reliability. The assumption was reliability of at least 0.70, thus imposing a constraint on the residual variance of the composite score for the specified latent variable. All outcome variables were included in the same model. The fit of this model was also excellent: χ^2^ = 421.64 (*df* = 202; *p* < 0.001), RMSEA = 0.052 (90% CI: 0.045, 0.059), CFI = 0.968, TLI = 0.937, SRMR = 0.21.

In order to place CQ and PWB in the nomological network of the SAPI, product-moment correlation analysis was used to determine the relationships between the SAPI and the constructs of CQ and PWB. The results are presented in Table 4. As can be seen from the table, CQ was most consistently correlated with PSR and Openness, with low to moderate correlations. The correlations with the other personality factors were generally smaller and limited to individual CQ components. The pattern is broadly consistent with previous research, although it appears that in the South African context, CQ can be best understood by its association with positive social-relational traits and openness. The limited correlations with extraversion and conscientiousness are different from some previous studies in Western samples and from Nel et al. (2015) results in South Africa using the early, conceptual SAPI model. These findings suggest that the broad PSR dimension may have subsumed some of the variance that could be attributed to other factors.

For the PWB factors, the most consistent and generally highest correlations were observed for Conscientiousness and Neuroticism, followed by Openness and the two social-relational factors. Consistently with previous research, Extraversion and PSR had their lowest correlation with Autonomy, and Openness had one of its lowest correlations with Environmental Mastery (Jones et al., 2015; Anglim and Grant, 2016). Also consistent with expectations, PSR and NSR were meaningfully related to the Relations with Other component of PWB. Finally, NSR tended to have meaningful associations with PWB, but was essentially not related to CQ.

### Study 2: The SAPI, Trait Anxiety, and Work Locus of Control

Study 3 extended the investigation into the convergent and discriminant validity of the SAPI and the establishment of its nomological network by first of all investigating the correlations between the SAPI and measures of anxiety and WLC.

Saviola et al. (2020) describe anxiety as “…a mental state characterised by an intense sense of tension, worry or apprehension, relative to something adverse that might happen in the future” (p. 1). Anxiety is usually studied either as a trait or a state (Wilt et al., 2011). Trait anxiety is a personality trait that can be identified as an individual’s general inclination to be anxious or the natural anxiety levels exhibited by a person (Vreeke and Muris, 2012; Leal et al., 2017). In contrast, state anxiety refers to a person’s anxiety levels over a short period without the presence of particular pathological conditions (Vreeke and Muris, 2012; Saviola et al., 2020). The present study focused on anxiety as a trait. Research has repeatedly shown that trait anxiety is positively related to the Big Five’s Neuroticism/Negative Emotionality (Kotov et al., 2007; Karsten et al., 2012; Vreeke and Muris, 2012; Watson and Naragon-Gainey, 2014; Fowler et al., 2017; Goldstein et al., 2018; Naragon-Gainey and Watson, 2018; Watson et al., 2019; Qu et al., 2020), and negatively related to Conscientiousness (Vreeke and Muris, 2012; Watson and Naragon-Gainey, 2014; Qu et al., 2020), and to Extraversion/Positive Emotionality (Kotov et al., 2007; Qu et al., 2020). Goldstein et al. (2018) found trait anxiety to be positively related to Openness, while Qu et al. (2020) found a significant negative relationship between the two constructs. In the HEXACO PI-R, Anxiety is a subscale of the Emotionality factor, suggesting that a person with very high scores on the Emotionality scale experiences anxiety in response to stressors (Lee and Ashton, 2004). Ashton et al. (2007) found that an Anxiety scale loaded strongly on low Agreeableness and Emotionality.

Locus of control refers to a person’s general level of expectancy toward a situation they have experienced (Aubé et al., 2007; Burger, 2008; Omari et al., 2012). People can present either an internal locus of control or an external locus of control. An internal locus of control refers to a person’s belief that the results of certain events are due to his or her personal ability, efforts, and dedication (Aubé et al., 2007; Omari et al., 2012). Individuals with an external locus of control tend to interpret events due to chance, luck, fate, authoritative others, or circumstantial complexity (Rotter, 1966; Van Praag et al., 2004; Burger, 2008; Aghaei et al., 2013). In an attempt to produce a work-specific measure of locus of control, Spector (1988) developed the Work Locus of Control Scale (WLCS) that measures generalised beliefs regarding whether or not people can control reinforcements within the work context, tapping into both internal control and external control (Spector and O′Connell, 1994; Oliver et al., 2006; Aubé et al., 2007). According to Bosman et al. (2005), a person’s assessment of the relationship between how they behave at work and the subsequent rewards or punishments represents that person’s work locus of control. External locus of control has been found to be positively correlated with Neuroticism and negatively correlated with the remainder of the Big Five factors (see Chen et al., 2016; Lovell and Brown, 2017; Smidt et al., 2018; Žitný and Halama, 2011). The more situation-specific external work locus of control trait has been positively correlated with psychological traits such as Neuroticism, Trait anxiety, work anxiety, Negative Affectivity, and Type A impatience (see Cook et al., 2000; Johnson et al., 2009), while negatively correlated with Extraversion, Agreeableness, Conscientiousness, emotional intelligence, Type A achievement, and autonomy (see Cook et al., 2000; Johnson et al., 2009; Spector and O′Connell, 1994).

Based on the previous research, it is expected that anxiety and external WLOC would be positively correlated with Neuroticism and would tend to have negative correlations with the rest of the SAPI factors except the Negative Social-Relational. As for the latter, because it shares aspects of negative valence with Neuroticism and is known to be moderately negatively correlated with the Positive Social-Relational factor (Fetvadjiev et al., 2015; Table 2), it can be expected to correlate positively with both criterion variables; the association with anxiety should be weaker than for Neuroticism, given the limited conceptual correspondence.

#### Method

The SAPI, a trait anxiety measure, and a work locus of control measure were administered to 422 adults from the general South African workforce in Sample B. Work locus of control was only assessed in the subsample of working adults.

We used the Generalised Anxiety Disorder 7-item scale (GAD-7), developed by Spitzer et al. (2006) to be used as a clinical measure to assess Generalised Anxiety Disorder. It can also be used to assess non-clinical trait anxiety. The GAD-7 is a seven-item scale that makes use of four response options, namely: “not at all” (0), “several days” (1), “more than half the days” (2), and “nearly every day” (3). No items are reverse scored. Scores on the GAD-7 range between zero and 21 and represent either mild (≥5), moderate (≥10) or severe (≥15) levels of symptoms of anxiety (Löwe et al., 2008). The Cronbach’s alpha coefficients of the GAD-7 suggests excellent internal consistency (0.92) (Spitzer et al., 2006).

To measure WLC, we used the Work Locus of Control Scale (WLCS) developed by Paul Spector (1988) to assess workplace beliefs that relate to both internal and external locus of control (Spector and O′Connell, 1994). The WLCS is a 16-item questionnaire rated on a six-point Likert scale ranging from 1 (disagree very much) to 6 (agree very much). Half of the questions/items relate to the internal rewards domain, and the other half relate to the external rewards domain (Spector and O′Connell, 1994). Therefore, half of the items (8 items) are reverse scored. In the interpretation of the WLCS, a high score is indicative of a more external locus of control, while a low score is indicative of a more internal locus of control (Macan et al., 1996; Aubé et al., 2007). The internal consistency of the WLCS coefficient alphas ranges from 0.75 to 0.85 (Spector, 1988; Spector et al., 2002). The Cronbach’s alpha coefficients of the SAPI, GAD-7, and WLCS in the current study are presented in Table 5.

**TABLE 5:** Correlations of the latent variables of Study 2.

	*M*	*SD*	α	1	2	3	4	5	6	7	8
Conscientiousness	4.04	0.75	0.92	–							
Extraversion	3.72	0.88	0.89	0.35	–						
Neuroticism	2.64	0.93	0.84	−0.30	−0.16	–					
Openness	3.99	0.73	0.89	0.51	0.49	−0.36	–				
NSR	2.01	0.89	0.89	−0.30	−0.05	0.29	−0.05	–			
PSR	3.91	0.68	0.95	0.58	0.56	−0.21	0.56	−0.30	–		
GAD	0.98	0.79	0.91	−0.14	−0.07	0.61	−0.17	0.17	−0.15	–	
WLC	2.49	0.68	0.84	−0.29	−0.17	0.22	−0.28	0.21	−0.30	0.21	–

*All displayed correlations below −0.07 and above −0.05 are significant at *p* < 0.05 or lower.*

*WLC, work locus of control; GAD, trait anxiety; NSR, Negative Social-Relational Disposition; PSR = Positive Social-Relational Disposition.*

#### Results

The six-factor model of the SAPI was fitted to the observed data using ESEM. The fit of the six-factor model proved to be excellent: χ^2^ = 217.81 (*df* = 72; *p* < 0.001), RMSEA = 0.069 (90% CI: 0.059, 0.080), CFI = 0.967, TLI = 0.921, SRMR = 0.019. Then an ESEM-within-CFA (set ESEM) method was used to add the factors into a new input file, using the same specification as described for Study 1. The fit of this model was also excellent: χ^2^ = 280.21 (*df* = 98; *p* < 0.001), RMSEA = 0.066 (90% CI: 0.057, 0.076), CFI = 0.961, TLI = 0.917, SRMR = 0.022.

The product-moment correlation between the SAPI, the WLCS, and the GAD-7 was examined to place Work Locus of Control and Trait Anxiety in the SAPI’s nomological network. Table 5 presents the correlations for the variables. In line with expectations, anxiety was strongly positively correlated with Neuroticism and had weaker correlations with the other factors. The sizes of WLC’s correlations varied little across factors and were also in line with previous research: External WLC was positively related to Neuroticism (as well as the Negative Social-Relational factor) and negatively to the other factors.

## General Discussion

The current study aimed to examine the psychometric properties of the SAPI (Fetvadjiev et al., 2015) by evaluating the internal structure and the external relations with various psychological traits. A newly developed instrument needs to demonstrate salient and consistent validity and reliability to be viewed as a dependable instrument in the relevant field (Ziegler et al., 2013). The SAPI model not only displayed consistent internal validity and consistency across three separate student and working adults samples, but also demonstrated meaningful relations with various psychological traits, adding to its nomological network.

The SAPI showed scalar measurement invariance pertaining to gender and ethnicity. Testing for measurement invariance plays a vital part in personality research since it is crucial to ensure that no elements are measured that may demonstrate bias and difference in meaning (Van de Vijver and Leung, 2021). Therefore, in this study, we did not detect bias in measuring personality based on gender and ethnicity, highlighting the potential of the SAPI as a bias-free instrument for cross-cultural comparisons.

The findings on the network of associations between the SAPI and relevant psychological traits are encouraging. We examined the correspondence of cultural intelligence, psychological well-being, work locus of control, and trait anxiety with the SAPI factors. We found that the SAPI factors correlated with various cultural intelligence factors on a small or medium effect, which is not surprising since the SAPI was developed by keeping in mind cultural and social factors, and the prominence of the social-relational orientation that was found in the initial phases of the SAPI development (Nel et al., 2012; Fetvadjiev et al., 2015). The strongest correlations seem to be between Openness with Meta-cognition CQ and Motivational CQ, while Positive Social-Relational Disposition corresponded strongly with Meta-cognition CQ. Both factors had associations with all CQ components It is evident that the cognitive adaptability in a multicultural context is associated with the traits of being open and managing constructive relations.

The SAPI factors correlated moderately to highly with psychological well-being factors. Conscientiousness (positively) and Neuroticism (negatively) corresponded strongly with all psychological well-being factors. Conscientiousness is associated with a person’s direction, organisation or positioning of life, while Neuroticism focuses on the emotional management of a person. Therefore, psychological well-being seems to relate strongly to elements captured in SAPI’s Conscientiousness and Neuroticism, in line with previous studies (Anglim et al., 2020; Ortet et al., 2020). Mastering and managing one’s environment and emotions are thus key qualities to obtain overall well-being. Another SAPI factor that correlated with all psychological well-being factors (except with Autonomy) is Positive Social-Relational Disposition. It is illustrative of the importance of the Positive Social-Relational Disposition that it correlated with the factors of subjective well-being since enhancing social relations is fundamental to achieve overall well-being (Anglim and Grant, 2016). Openness, in turn, showed strong correspondence with Personal Growth. Personal growth is close to the meta-cognition process of cultural intelligence, which corresponded closely with SAPI’s Openness. This further enforces the relation that a person needs to demonstrate Openness to manage self-relation and how one views the social environment and process information for appropriate conduct. This finding highlights the practical relevance of Openness, a factor whose replication has sometimes been questioned in etic studies where Western instruments have been used in non-Western populations. Taken as a whole, our findings illustrate the strong potential of the SAPI to predict individual differences in elements important to well-being.

In line with expectations, Neuroticism correlated strongly with trait anxiety, which confirms previous findings (Fowler et al., 2017; Goldstein et al., 2018; Naragon-Gainey and Watson, 2018; Watson et al., 2019; Qu et al., 2020). Finally, work locus of control correlated with most factors of the SAPI, particularly with Conscientiousness, Openness, and Positive Social-Relational Disposition. The understanding of work locus of control pertains to the internal and external control of elements in the workplace and the outcomes thereof (Oliver et al., 2006; Aubé et al., 2007). Whereas Positive Social-Relational Disposition pertains more to the person’s constructive handling and managing of relationships with others (Fetvadjiev et al., 2015), work locus of control may be partly related the tendency to maintain positive relations, in whether a person can control aspects of the social work environment to facilitate constructive relations.

## Limitations

The current study is not without limitations. The cross-sectional nature of the various studies implies that causal relationships between the various psychological constructs and the SAPI could not be determined. Longitudinal studies could identify trends and changes in the relationship between personality and these constructs over time. Another limitation is the lack of an appropriate sample size for the Indian ethnic group; it is recommended that a more stratified approach be taken in future SAPI research to ensure equal representation of the various race groups, and that overall larger samples are studied. Considering the high correlation of neuroticism with anxiety in the current study, it is also interesting to explore the potential application of the SAPI in clinical samples.

## Conclusion

This study generated promising results to support the saliency of the SAPI factors. Finding an appropriate internal structure that measures personality without bias in a diverse context is not easy, and this study provided strong evidence that the SAPI is on the right track to be a dependable and sound personality instrument. The SAPI factors had meaningful associations with relevant psychological outcomes. This study adds to the growing field of emic–etic personality research by embedding the indigenously derived SAPI in a broader network of previously established psychological traits. Future emic–etic research should seek to further examine the nomological networks of indigenous personality measures with reference to both universal and locally salient psychological outcomes.

## Data Availability Statement

The datasets generated during and analysed during the current study are not publicly available due to the copyrighted items. Requests to access the datasets should be directed to CH, chill@uj.ac.za.

## Ethics Statement

The studies involving human participants were reviewed and approved by Department of Industrial Psychology and People Management, Research Ethics Committee, University of Johannesburg and WorkWell Research Unit Research Ethics Committee, North-West University. The participants provided their written informed consent to participate in this study.

## Author Contributions

CH and JN contributed to conception and design of the study. CH, LS, and MB organised the database. LB performed the statistical analysis. CH, LS, and MB wrote the first draft of the manuscript. CH, JN, LB, and VF wrote sections of the manuscript. CH, JN, LB, and VF contributed to manuscript revision, read, and approved the submitted version. All authors contributed to the article and approved the submitted version.

## Conflict of Interest

The authors declare that the research was conducted in the absence of any commercial or financial relationships that could be construed as a potential conflict of interest.

## Publisher’s Note

All claims expressed in this article are solely those of the authors and do not necessarily represent those of their affiliated organizations, or those of the publisher, the editors and the reviewers. Any product that may be evaluated in this article, or claim that may be made by its manufacturer, is not guaranteed or endorsed by the publisher.

## Footnotes

1 An official South African term used for people from African descent.

2 An official South African term used for people from mixed descent.
